# Complete Genome Sequences of Five Bluetongue Virus (BTV) Vaccine Strains from a Commercial Live Attenuated Vaccine, a BTV-4 Field Strain from South Africa, and a Reassortant Strain Isolated from Experimentally Vaccinated Cattle

**DOI:** 10.1128/genomeA.00462-16

**Published:** 2016-06-23

**Authors:** Carien van den Bergh, Peter Coetzee, Alan J. Guthrie, Misha le Grange, Estelle H. Venter

**Affiliations:** Department of Veterinary Tropical Diseases, Equine Research Centre, Faculty of Veterinary Science, University of Pretoria, Onderstepoort, South Africa

## Abstract

This is a report of the complete genome sequences of plaque-selected isolates of each of the five virus strains included in a South African commercial trivalent bluetongue virus (BTV) attenuated live virus vaccine, a BTV-4 field strain isolated from Rustenburg, South Africa, in 2011, and a bluetongue reassortant (bluetongue virus 4 strain 4/*O. aries*-tc/ZAF/11/OBP-115) isolated from experimentally vaccinated cattle. Full-genome sequencing and phylogenetic analyses show that the bluetongue virus 9 strain 9/*B. taurus*-tc/ZAF/15/Onderstepoort_B02b is a reassortant virus containing segments from both BTV-9 and BTV-8.

## GENOME ANNOUNCEMENT

Bluetongue virus (BTV) is a noncontagious disease of ruminants that is transmitted by *Culicoides* biting midges (Diptera: Ceratopogonidae) (1) (genus *Orbivirus*, family *Reoviridae*). The genome of BTV is composed of 10 segments of double-stranded RNA (dsRNA) that collectively encode seven structural proteins and five nonstructural proteins (2–4). In South Africa, a polyvalent BTV-modified live virus (MLV) vaccine is manufactured by Onderstepoort Biological Products (OBP) Ltd. This vaccine is supplied in three separate vials, each of which contains different BTV serotypes: bottle A contains serotypes 1, 4, 6, 12, and 14, bottle B contains serotypes 3, 8, 9, 10, and 11, while bottle C contains serotypes 2, 5, 7, 13, and 17. Here, we report the full-genome sequences of BTV-3, BTV-8, BTV-9, BTV-10, and BTV-11, which were isolated from bottle B of the BTV-MLV vaccine (batch 115; OBP Ltd., Onderstepoort, South Africa), a BTV-4 field strain isolated from a clinically infected ewe in Rustenburg, and a reassortant strain between BTV-9 and BTV-8 segment 8 isolated from experimentally vaccinated cattle. The individual serotypes were independently isolated from the vaccine bottle and/or infected blood using plaque selection on Vero cells (5). Each of these viruses was then passaged on monolayers of Vero African green monkey cells grown in 25-cm^2^ tissue culture flasks. BTV dsRNA was extracted from virus-infected cells using TRIzol reagent (Life Technologies, Johannesburg, South Africa). Sequencing templates were prepared using full-length amplification of cDNAs (6). Amplicons were sequenced on an Illumina MiSeq sequencer (Inqaba Biotechnical Industries [Pty] Ltd., Pretoria, South Africa) using the Nextera XT DNA sample preparation kit and 300-bp paired-end V3 Illumina chemistry. The Illumina sequence reads were analyzed using Geneious 8.1.5. A combination of *de novo* assembly, followed by mapping to reference genomes available on GenBank, was used to obtain the full-length genome sequences of the seven viruses.

Pairwise nucleotide sequence identities between the genome sequences derived from the isolated BTV-MLVs and MLV sequences already available on GenBank were as follows: bluetongue virus 3 strain3/Labstr/ZAF/14/OBP-115 was 99% identical to BTV-3 isolate RSArrrr/03, bluetongue virus 8 strain8/Labstr/ZAF/14/OBP-115 was 99% identical to BTV-8 isolate RSArrrr/08, bluetongue virus 9 strain9/Labstr/ZAF/14/OBP-115 was 99 to 100% identical to BTV-9 isolate RSArrrr/09, bluetongue virus 10 strain10/Labstr/ZAF/14/OBP-115 was 99% identical to BTV-10 isolate 2627, and bluetongue virus 11 strain11/Labstr/ZAF/14/OBP-115 was 99% identical to BTV-11 strain BTV-11 VAC.

### Nucleotide sequence accession numbers.

The full-genome sequences of bluetongue virus 3 strain 3/Labstr/ZAF/14/OBP-115, bluetongue virus 8 strain 8/Labstr/ZAF/14/OBP-115, bluetongue virus 9 strain9/Labstr/ZAF/14/OBP-115, bluetongue virus 10 strain10/Labstr/ZAF/14/OBP-115, and bluetongue virus 11 strain11/Labstr/ZAF/14/OBP-115 have been deposited in GenBank under the accession numbers KT317675 to KT317684, KT317685 to KT317694, KT885055 to KT885064, KT317695 to KT317704, and KT885065 to KT885074, respectively. The full-genome sequences of the wild-type strain of BTV-4 (bluetongue virus 4 strain 4/*O. aries*-tc/ZAF/11/OBP-115) are available on GenBank as accession numbers KT317665 to KT317674. The full-genome sequences of the bluetongue virus 9 strain 9/*B. taurus*-tc/ZAF/14/Onderstepoort_B02b that is a reassortant between BTV-9 and BTV-8 has been deposited in GenBank as accession numbers KT885075 to KT885084.
